# Application of the varying coefficient model to the behaviour risk factor surveillance data in Italy: a study of changing smoking prevalence among sub-populations

**DOI:** 10.1186/s12889-015-1805-3

**Published:** 2015-05-13

**Authors:** Shireen Assaf, Stefano Campostrini

**Affiliations:** Department of Statistical Sciences, University of Padova, Padova, Italy; ICF International, Rockville, USA; Ca’ Foscari University, Venice, Italy

**Keywords:** P-spline, Big data, PASSI, Odds ratio plots, Health promotion policy and evaluation

## Abstract

**Background:**

Behaviour risk factor surveillance (BRFS) data can be an important source of information for studying changes in various health outcomes and risk factors. Results obtained from surveillance data analysis are vital for informing health policy interventions, particularly with regards to evolutionary aspects. The objective of this analysis was to recommend a method that can be used for analysing trends in the association among variables from large public health data sets. This was demonstrated by examining the changing effects of various covariates, representing different sub-populations, on smoking status over time.

**Methods:**

In our work, we propose the use of varying coefficient models (VCM) with non-parametric techniques to catch the dynamics of the evolutionary processes under study. This is a useful method, which allows coefficients to vary with time using smooth functions. Italian BRFS data from 2008-2012 was used with a sample size of 185,619 observations. In the application, a time VCM is fit for a smoking status binary outcome variable using the P-spline estimation method. The model includes ten independent variables comprising socio-demographic, health risk and behaviour variables.

**Results:**

The VCM fit for the data indicates that the coefficients for some of the categories for the age and the alcohol consumption variables varied with time. The main results show that Italians aged 18-29 and 40-49 had higher odds of being smokers compared to those aged 60-69; however, these odds significantly decreased in the period 2008-2012. In addition, those who do not drink had lower odds for being a smoker compared to high risk drinkers and these odds decreased further during the observation period.

**Conclusion:**

The application of the VCM to the BRFS data in Italy has shown that this method can be useful in detecting which sub-populations require interventions. Although the results have shown a decrease in the odds of being a smoker for certain age groups and non-drinkers, other sub-populations have not decreased their odds and health inequalities remain. This observation indicates that efforts and interventions are still required to target these non-changing sub-populations in order to modify their smoking behaviour.

## Background

Public health surveillance systems have a long history, starting with the collection of data for monitoring infectious diseases to the study of non-communicable diseases in more recent years [1]. The data collected can vary from one country to another, where some collect detailed health risk and behaviour information related to non-communicable disease such as the United States of America Behavioural Risk Factor Surveillance System (US BRFSS), and the similarly designed *Progressi delle Aziende Sanitarie per la Salute* (PASSI) Italian surveillance system. The common characteristic of all surveillance systems is to have a continuous data collection system [2] usually by taking a new and independent random sample at each time period and therefore individuals are not followed as in longitudinal studies. Some surveillance systems collect data more frequently than others do. The US BRFSS system, for example, has been collecting data since 1984, where a new random sample is taken each month for a telephone interview [3]. The Italian PASSI surveillance system, initiated in 2007, also collects data on a monthly basis and is ongoing. Therefore, these types of surveillance systems are producing data that are continuously increasing in sample size. Surveillance systems can be considered to be another form of big data which is proving to be an important source of information, particularly when statistical well-thought methods [4] can take advantage of the availability of this rich data, which is very informative especially for evolutionary analysis.

More specifically, the analysis of Behaviour risk factor surveillance (BRFS) data can be very useful for examining trends of health outcomes and risk factors since these data are collected for long periods of time in an almost continuous fashion. Although often a simple time trend analysis answers many possible questions (e.g. is smoking increasing or decreasing?), sometimes the substantial questions are much more complex and ask for more sophisticated models. This is usually the case when wanting to answer the question “why is there a change?” or examining how the relationships among some variables are changing over time. The richness of BRFS data could offer such information [5], although, so far, rarely has it been properly addressed with sufficiently advanced statistical approaches. In the traditional trend analysis methods, such as in time series analysis, observations are assumed to be dependent and need to be aggregated before analysis, for instance to analyse changes over time in means or proportions. The aggregation of data can cause some loss of information on the variability between the observations, since it typically uses only monthly means. The assumption of dependence requires the model to be reformulated in order to take into account the dependence structure in the data. However, in BRFS data the samples are taken randomly each month and therefore the observations can be assumed to be independent. In addition, the usual methods used for the analysis of trends assume that each variable has one parameter to be estimated for the entire period of observation; therefore assuming that the parameters are constant with time. The proposed method of using varying coefficient models (VCM) for analysis of surveillance data allows one variable to vary by another therefore creating coefficients that vary with the modifying variable, which can be time. The method also does not require the aggregation of observations and does not require the assumption of dependence between the observations. The VCM can provide a flexible model for studying the dynamic and evolutionary BRFS data, and can be used to answer a different type of question in trend analysis. This question is not specifically concerned with studying the trends of the outcome, but in studying the trends in the effects (i.e. the coefficients), which can give us a deeper understanding of the changes in the subgroups of the population with respect to the outcome of interest. These models are well established in the literature and their use for the study of trends has been demonstrated by many authors, however with the use of longitudinal data [6-11]. While Young *et al.* [12] used the US BRFSS surveillance data as well as other environmental data for fitting a spatial varying coefficient model for the study of the spatial variation of the effect of ozone levels on myocardial infraction occurrence. However, to the best of our knowledge, the use of varying coefficient models for studying time varying coefficients using behaviour risk factor surveillance data, or similar health related big data, has not yet been performed. Therefore, there is potential to utilize this data even further which will be demonstrated in this analysis.

The aim of our study is to evaluate the applicability of the varying coefficient model approach to BRFS data. First to examine its feasibility from a computational point of view due to the growing sample size of these data collection systems, but more importantly, its relative capability of offering possibly more informative and readable results. The method was applied to studying smoking status in Italy as the outcome of interest.

## Data and methods

### Description of the data

The data used for the analysis is from the PASSI (Progressi delle Aziende Sanitarie per la Salute or Progress in the Italian Local Health Units) surveillance system in Italy [13], the details of which are described by Baldissera *et al.* [14], as well as Minardi *et al.* [15] and Binkin *et al.* [16], who have used the PASSI data for analysis of health outcomes and risk factors. PASSI raw data are not openly available outside the PASSI network; however, their use for research purposes is encouraged once permission is obtained from the National Coordinating Group at the Italian National Institute of Health (Istituto Superiore di Sanità). No experiments involving human subjects was performed for the PASSI surveillance system and approval for the PASSI was provided by the Italian National Health Institute ethical board. Data collection for PASSI is conducted through the local health units. These units are found in all the 21 Italian regions and comprise the basic structure of the National Italian Health System (a public universal health system). Each region in Italy has between 1 and 22 local health units. The coverage for the data collection are all registered users of the participating local health unit. PASSI data collection began in 2007 and is still ongoing and is conducted by each local health unit participating in the surveillance system, which is over 90*%* of the Italian local health units. To collect the data, a monthly random sample with replacement is chosen from a list of residents in each local health unit aged 18 - 69 years and a telephone interview is conducted with those selected. The sample selection is stratified based on an age and sex distribution weighting system in order to adjust for the different population sizes of the local health units. Each month a new random sample is selected from the list of residents, therefore the same individuals are not followed over time as in longitudinal studies.

The questionnaire used in the telephone interview covers a wide variety of behavioural and preventive topics, and the same questionnaire is used in all the Italian regions. The wording of the questions has been kept relatively constant over the years except for a few variables not included in the analysis. Any slight change in the wording of the questions has been taken into account before creating the variables required for analysis. For performing the analysis, the monthly data are combined for all the years from 2008 to 2012.

For the analysis, eleven variables are constructed from the data, one response variable and ten independent variables. The smoking status binary response variable was constructed from an existing smoking variable with four categories. For the binary variable, current smokers status, the categories of ‘smoker’ and ‘persons attempting to quit’ were combined, while ‘non-smokers’ combined the categories of ‘non-smoker’ and ‘ex-smoker’. The independent variables are chosen based on what has been indicated in the literature review to be possibly associated with smoking and smoking cessation. These included socioeconomic or socio-demographic variables [17-21], as well as health risk and behaviour variables [22-25]. The ten independent variables used in the analysis are age, sex, marital status, education level, income level (or economic difficulties), work status, and region to represent the socioeconomic and socio-demographic variables, and alcohol consumption, physical activity and depression status to represent the health risk and behaviour variables. See Table 1 for a description of these variables and their categories. Most of these variables are self-explanatory however, the health risk and behaviour variables have specific definitions. For the alcohol consumption variable, non-drinkers are those that indicated they did not have at least one drink in the last 30 days when interviewed. High risk drinkers are those that drink on average per day more than one unit of alcohol if the subject is a female and more than two units of alcohol if the subject is a male. If the subject drinks less than this amount then they are classified as a low risk drinker. An alcoholic unit corresponds to 12 grams of ethanol, which is the amount found in approximately one can of beer (330 ml), a glass of wine (125 ml) or a shot of liquor (40 ml). The physical activity variable was constructed from questions asking about the subject’s physical activity during their free time and at work which resulted in three categories: active, partially active and sedentary. An active person is considered a person who performs heavy work or has a job that requires a lot of physical effort, or who performs moderate physical activity for at least five days a week for 30 minutes, or performs vigorous activity at least three days a week for more than 20 minutes. A partially active person is a person who does not have a heavy physical job but still does some physical activity in their free time, without reaching the recommended physical activity guideline levels. A sedentary person is a person who does not have a heavy physical job and also does not exercise in their free time. Finally, the depression status binary variable was constructed from questions asking about the subject’s morale and feelings of depression, and was constructed following the technique of Binkin *et al.* [16] who have also used PASSI data for studying depression. In addition to these ten independent variables, the time variable was constructed from the month and year in which the interview was conducted for each observation.

**Table 1:** **Description of variables used in the analysis for the period between 2008-2012**

**Variable**	**Categories**	**Number of**	**%**
		**observations**	
Smoking status	Smoker	51696	27.9
	Non-smoker	133923	72.1
Age	18-29	33450	18.0
	30-39	38660	20.8
	40-49	44193	23.8
	50-59	35646	19.2
	60-69	33670	18.2
Sex	Male	91160	49.1
	Female	94459	50.9
Marital status	Married	112853	60.8
	Single	57959	31.2
	Widowed or divorced	14807	8.0
Education	University or higher	24700	13.3
	High school	82324	44.4
	Middle school	58063	31.3
	Primary school or less	20532	11.0
Income	High	87989	47.4
	Medium	74159	40.0
	Low	23471	12.6
Work status	Works	107648	58.0
	Does not work	77971	42.0
Region	North	92741	50.0
	Central	45142	24.3
	South	47736	25.7
Physical activity	Active	61357	33.1
	Partially active	70800	38.1
	Sedentary	53462	28.8
Alcohol consumption	High risk drinker	18852	10.2
	Low risk drinker	52838	28.4
	Non-drinker	113929	61.4
Depression status	Not depressed	173416	93.4
	Depressed	12203	6.6
Year	2008	37205	20.0
	2009	38690	20.8
	2010	35896	19.2
	2011	36825	19.8
	2012	37003	20.0

### Methods

For the analysis of the data, a time-varying coefficient model was found using P-spline estimation which requires indicating the degree of the spline (usually a B-spline) as well as the degree of the difference penalty (the difference of the adjacent B-spline coefficients) to perform the computation. Following the recommendations of Eilers and Marx [26], a third degree B-spline is used with a second order difference penalty. This method also requires placing a large number of knots to purposefully overfit the data so that the penalty does the work of regulating the smoothness of the coefficient functions. Below is a brief presentation of the estimation method used followed by the description of the methodology and application used to fit the time-varying coefficient model for smoking status outcome in Italy.

#### Model and estimation method

One of the first articles to discuss varying coefficient models by Hastie and Tibshirani [27] shows how these models essentially contain a coefficient which is a function of a variable, and these functions can either be flexible parametric functions such as Fourier series or piecewise polynomials, or more generally non-parametric functions. The simplest form of the varying coefficient model is the the Gaussian model where we have *Y* as a normally distributed dependent variable with mean *μ* [27]. This model has the form

(1) $$ Y = b_{0} + \sum_{j=1}^{p} b_{j} Z_{j} + a_{0}(t) + X_{1} a_{1}(t) + \ldots + X_{p} a_{p}(t) + \epsilon  $$

where *E*(*ε*)=0 and *var*(*ε*)=*σ*^2^ [27]. The covariates are **Z**=(*Z*_1_,…,*Z*_*p*_)^*T*^ with the constant coefficients *b*_*j*_ and **X**=(*X*_1_,…,*X*_*p*_)^*T*^ with the varying coefficients *a*_*j*_. The intercepts are represented by *b*_0_ and *a*_0_, where *b*_0_ is the constant intercept and *a*_0_ is the time varying intercept. The effect modifier covariate *t* represents time; however, the effect modifier can technically be any covariate or multiple covariates and it is the variable that is used to express the coefficients as a function. In this case, the coefficients are a function of time. This model is referred to by Hastie and Tibshirani [27] as a varying coefficient model with a single effect modifier and can also be referred to as a time-varying coefficient model. In BRFS data the time variable represents the time of observation. In longitudinal data, the age of the respondent can also represent the time effect modifier, since the same observation is followed over time.

The VCM can be extended to the non-Gaussian cases, i.e. for a generalized varying coefficient model, through a link function *g*(*μ*) as in generalized linear models. In the generalized varying coefficient model, we have a random variable *Y* with a distribution which depends on a parameter *η* which is a linear predictor related to the mean *μ*=*E*(*Y*) by *η*=*g*(*μ*) [27]. In the Gaussian case, the normally distributed random variable *Y* has a mean *μ*=*η*=*g*(*μ*). Another common model is the case where *Y* is a binary variable and the model is a logistic model with $g(\mu)= \log \left \{\frac {\mu }{(1-\mu)}\right \}$. This is an especially common case in the analysis of health data in which the response variables are usually binary variables.

Researchers studying the varying coefficient model have mainly used non-parametric methods with two main estimations techniques: kernel-local polynomial smoothing and estimation using splines (polynomial spline, penalized regression spline or smoothing spline). Kernel-local polynomial estimation was used by Cheng *et al.* [28] for estimating a varying coefficient model with a binary response variable of infant mortality, and Cai *et al.* [29,30] applied the technique to Poisson and binary response variables. Smoothing spline estimation was first described by Hastie and Tibshirani [27] and was used by Hoover *et al.* [7] and Chiang *et al.* [31] for longitudinal data analysis; polynomial and penalized regression estimation have been discussed by Huang *et al.* [32] and Marx [33]. The use of parametric methods for estimating varying coefficient models are not favoured for estimation due to the lack of flexibility of these methods as well as the strong assumptions required which can lead to misspecification of the data and large bias [27,34]. The analysis in this paper uses non-parametric techniques with the PASSI surveillance data to find a generalized time-varying coefficient model. P-spline estimation was selected for estimation mainly due to the flexibility it provides the user as discussed herin and its reduced computation time compared to other methods considered (results not shown). However, other spline or kernel methods can also be used.

Splines are merely functions joined together at certain positions (or knots), and the different techniques for estimation using splines depend on the method used to regulate the smoothness of the functions. In polynomial spline estimation, there is no penalty and smoothness is regulated by selecting the ideal number of knots using a certain criteria such as the Akaike information criteria (AIC). This selection of the number of knots can greatly increase computation time as several models need to be fit with different number of knots in order to select the model which provides the minimum AIC.

In smoothing spline and penalized regression spline estimation, a penalty is added to regulate the smoothness of the spline after placing a sufficiently large number of knots. For instance, beginning with the model *y*_*i*_=*x*_*i*1_*a*_1_(*t*)+…+*x*_*ip*_*a*_*p*_(*t*)+*ε*_*i*_ from the observations *y*_1_,…,*y*_*n*_, *x*_*ij*_ from the predictor *X*_*j*_ and *t* as the time effect modifier, in smoothing spline estimation the following is minimized to find the estimates, [27].

(2) $${} \lambda_{j}\! \int \!a_{j}^{\prime\prime}(u_{j})^{2} {du}_{j} \sum_{i=1}^{n} \!\left\{y_{i} -\! \sum_{j=1}^{p} x_{ij} a_{j} (t) \right\}^{2} + \sum_{j=1}^{p} \lambda_{j} \int a_{j}^{\prime\prime}(t)^{2} {dt}_{j}  $$

The first term is the summation of the square residuals and the second term is the summation of the penalties for each coefficient function. The smoothing parameter, *λ*_*j*_, penalizes the roughness of the coefficients *a*_*j*_ [27]. To find the estimates, the coefficient functions *a*_*j*_() can be expressed in terms of basis functions

(3) $$ a_{j}(t) = \sum_{l=1}^{n_{j}} \gamma_{ij} B_{jl}(t),  $$

where *n*_*j*_ are the number of unique values of *t* (i.e. the number of knots). The *B*_*jl*_(*t*) are basis functions for the *j*^*th*^ variable with parameters *γ*_*ij*_, and can be polynomial bases, natural cubic splines or B-splines functions [27].

In P-spline estimation (one type of penalized regression spline estimation), a different type of penalty is used which is constructed by placing a difference penalty on the coefficients of adjacent B-splines. The main advantages of using P-splines, as described by Eilers and Marx [35], are that P-splines have no boundary effects and they conserve moments of the data. The use of B-splines as basis functions is favoured in the literature due to their good numerical properties, as they have compact support that can speed up calculations [36,37]. To write the penalized sum of squares for using P-spline estimation in matrix form, let **a**_*j*_ represent *a*_*j*_(*t*) evaluated at the *n* observed values of *t* and **B**_*j*_ a matrix of spline functions so that equation (3) can be written as **a**_*j*_=**B**_*j*_***γ***_*j*_, where ***γ***_*j*_ is a vector of the basis parameters. Then the following is minimized to find the estimates

$$\bigg\Vert \mathbf{y} - \sum_{j=1}^{p} \mathbf{D}_{j} \mathbf{B}_{j} \boldsymbol{\gamma}_{j} \bigg\Vert^{2} + \sum_{j=1}^{p} \lambda_{j} \parallel \boldsymbol{\Delta}_{d} \boldsymbol{\gamma}_{j} \parallel^{2}, $$

where **D**_*j*_ is the diagonal matrix with the *n* observed values of *X*_*j*_. The penalty terms are represented by ***Δ***_*d*_, which is a matrix which constructs the *d*^*th*^ differences of ***γ*** or the difference penalty [26,33,35]. The *λ* parameter is the smoothing parameter found by cross-validation, generalized cross validation or also restricted maximum likelihood (REML) methods [38].

By defining **R**=**D**_*j*_**B**_*j*_, the above loss is minimized to find $\boldsymbol {\hat {\gamma }}$ by

$$\hat{\boldsymbol{\gamma}} = \left(\mathbf{R}^{T} \mathbf{R} + P \right)^{-1} \mathbf{R}^{T} \mathbf{Y}. $$

The matrix $P = block diag(\lambda _{0} \boldsymbol {\Delta }_{d}^{T} \boldsymbol {\Delta }_{d}, \ldots, \lambda _{p} \boldsymbol {\Delta }_{d}^{T} \boldsymbol {\Delta }_{d})$ has a block diagonal structure that breaks the linkage of the penalization from one smooth term to the next. The penalty in P-spline estimation can have different degrees so that the first, second or third difference can be taken. Eilers and Marx [26] recommend using at least a second degree difference penalty with either a quadratic or cubic B-spline basis. Therefore, the P-spline estimation method allows more flexibility as it allows the selection of the degree of the penalty and the spline separately which cannot be done using other spline estimation methods. In addition, the difference penalty described above can be considered simpler and less computationally intensive compared to the smoothing spline penalty shown in (2).

The adaptation of the P-spline to a generalized varying coefficient model was described by Eilers and Marx [26] and Marx [33] and this simply involves the maximization of the penalized log-likelihood

$$l(\boldsymbol{\gamma}) - \sum_{j=1}^{p} \lambda_{j} \parallel \boldsymbol{\Delta}^{d} \boldsymbol{\gamma}_{j} \parallel^{2}, $$

where *l*(***γ***) is the log-likelihood function. Here the penalty term is subtracted from the log-likelihood function to discourage roughness of any varying coefficient vector, and a Fisher’s scoring algorithm is used to find the estimates.

#### Application: fitting the time VCM

A varying coefficient model is applied to the Italian PASSI data for the period from 2008 to 2012 which provided a sample size of 185,619 individuals. The outcome of interest is smoking status which is a binary response variable to indicate current smokers and non-smokers, where non-smokers includes ex-smokers. Using ten independent variables constructed from the data, the effects of these variables are examined to observe their trends with time and understand the changing characteristics of smokers during this period (see Table 1 for a description of the variables used in the analysis).

For the period of observation used in the analysis (from 2008 to 2012) there are 55 months in which data collection was performed, as July and August were combined in each year in the data collection phase. These 55 months represent the effect modifier of time in the analysis which is constructed from the month and year of the survey data for each observation. They also represent the maximum number of knots that can be used in the estimation, and which is the number of knots used in this application. It is not required or advisable to place the maximum number of knots for very large datasets as this will greatly increase computation times for fitting the models. However, the computation time of the present application allowed for placing the maximum number of knots. This did not affect the results as the presence of the penalty term controls for any over-smoothing.

The first step in using the polynomial estimation method is to find whether each independent variable contains coefficients that are significantly changing over time, since there are coefficients which may be constant over time. Therefore, each independent variable is fit with the response variable, smoking status, and the independent variable is allowed to have varying coefficients while the other variables are kept constant. This model is then tested against the parametric model to see if the coefficients are actually varying. In other words, the following alternative hypothesis is tested against the parametric null hypothesis or logistic model in this case, i.e.

$$\begin{array}{*{20}l} \mathbf{H_{0}}: ~ \text{logit}(SMK) & = \sum_{j=1}^{p} b_{j} Z_{j}, \\ \mathbf{H_{1}}: ~ \text{logit}(SMK) & = \sum_{j=1}^{p} b_{j} Z_{j} + a_{1}(t)X_{1}, \end{array} $$

where $\text {logit}(SMK) = \log \frac {\Pr (Y = SMK)}{1 - \Pr (Y=SMK)}$ or the log of the odds of being a smoker (SMK). The *Z*_*j*_ are the constant variables from the parametric model with constant coefficients *b*_*j*_, and *a*_1_(*t*) is the varying coefficient of *X*_1_. Note that the variable *X*_1_ is contained in *Z*_*j*_ with its constant coefficient *b*_*j*_. This test would show if the varying coefficients *a*_1_(·) are actually varying or should remain constant, and it is performed using a likelihood ratio test with a chi-square distribution. This test can be used since there are a limited number of parameters to be estimated (i.e. the number of parameters and not increasing with increasing sample size), and the sample size is large enough to guarantee the asymptotic chi-square distribution of the test statistics. All tests and estimations were performed at the 95% confidence level using R statistical software as described further in the Endnote.

The final step involves finding the full varying coefficient model when more than one variable with varying coefficients are required in the model. This is conducted using a stepwise forward selection method beginning with the model which contains the most significant varying coefficient and then testing for the addition of each new varying coefficient. The selection of the variable to add next was determined by which variable provided the highest deviance explained in a model fit with the residuals of the previous model and each of the variables separately. The stepwise building of the model involved testing the following hypotheses:

$$\begin{array}{*{20}l} \mathbf{H_{0}}: ~ \text{logit}(SMK) & = \sum_{j=1}^{p} b_{j} Z_{j} + a_{1}(t)X_{1},\\ \mathbf{H_{1}}: ~ \text{logit}(SMK) & = \sum_{j=1}^{p} b_{j} Z_{j} + a_{1}(t)X_{1} + a_{2}(t)X_{2}, \end{array} $$

where *H*_0_ is the null hypothesis for the model which contains the first varying coefficient and *H*_1_ is the alternative hypothesis for the addition of a second varying coefficient for the variable *X*_2_. The variable *X*_2_ would also have been used in the first test to see if it has coefficients that are actually varying. Therefore, only the variables which were found to have varying coefficients when tested alone would be considered for testing in the stepwise selection process.

## Results

### Smoking time-varying coefficient model

Following the first step of the methodology, a model is fit where each independent variable is allowed to have varying coefficients and this model is then tested against the parametric model to see if the coefficients are actually varying. As shown in the first part of Table 2, all the tests performed for each variable separately presented a significant p-value and therefore were found to have varying coefficients. In addition to the p-values of the tests, the AIC of each model is reported and each model was found to have a lower AIC than the logistic model. The next step involves building of the model and testing whether the varying coefficients of one variable are still required when another is already present. The results of this step are shown in the second part of Table 2, in which p-values which are significant at the 0.05 significant level are indicated in bold. In this step, the results indicate that only two of the independent variables, age and alcohol consumption, have varying coefficients while all the other coefficients for the remaining independent variables are constant. This result was also found when observing the AIC values of each model. As shown in Table 2, Model I which contains varying coefficients for age and alcohol variables was found to have a lower AIC than the model which contains age with time varying coefficients. Although Model II had a slightly lower AIC than Model I (decrease of approximately 1.1), the test for the inclusion of the physical activity variable with varying coefficients was not significant and therefore this model was not selected. Model X (which is similar to Model I but with a time varying intercept) was selected due to significant p-values of the test. The AIC of this model was approximately equal to that of Model I. It is important to note that regional effects were taken into account and were found to have non-significant varying coefficients.

**Table 2:** **Fitting the smoking status time-varying coefficient model**

**Model**	**Description**	**Time (min)**	**p-value**	**H**_**0**_ ** used**	**AIC**	**df**
			**of test**	**for test**		
**Selection of variables that have varying coefficients**						
LM	logistic model	<1	-	-	206590.4	21.00
Model age	LM + s(t):age	4.7	**<0.001**	LM	206554.4	28.88
Model alcohol	LM + s(t):alcohol use	2.1	**<0.001**	LM	206566.0	27.39
Model physical	LM + s(t):physical activity	2.1	**<0.001**	LM	206570.1	26.19
Model income	LM + s(t):income	2.2	**<0.001**	LM	206574.8	26.33
Model mstatus	LM + s(t):martital status	2.1	**<0.001**	LM	206573.8	26.50
Model edu	LM + s(t):education	3.1	**<0.001**	LM	206573.2	26.26
Model sex	LM + s(t):sex	1.4	**<0.001**	LM	206572.4	25.37
Model work	LM + s(t):work status	1.4	**<0.001**	LM	206575.2	25.08
Model region	LM + s(t):region	2.2	**<0.001**	LM	206573.2	26.81
Model depress	LM + s(t):depression status	1.4	**<0.001**	LM	206574.2	24.49
Model time	LM + s(t)	1.6	**<0.001**	LM	206572.7	23.72
**Finding the full varying coefficient model**						
Model I	Model age + s(t):alcohol use	8.9	**0.008**	Model age	206548.7	33.21
Model II	Model I + s(t):physical activity	16.8	0.070	Model I	206547.6	36.40
Model III	Model II + s(t):income	25.7	0.261	Model II	206549.0	38.45
Model IV	Model II + s(t):marital status	24.4	0.539	Model II	206550.8	38.72
Model V	Model II + s(t):education	36.5	0.227	Model II	206549.4	39.48
Model VI	Model II + s(t):sex	24.4	0.125	Model II	206547.3	37.34
Model VII	Model II + s(t):work status	21.4	0.550	Model II	206549.3	27.38
Model VIII	Model II + s(t):region	26.5	0.470	Model II	206550.0	38.32
Model IX	Model II + s(t):depression status	22.0	0.369	Model II	206548.9	37.42
Model X	Model I + s(t)	10.4	**0.006**	Model I	206548.7	33.21
**Notes: s(t) - spline of time**						

The time required to fit the models was relatively short (in comparison with other approaches attempted - results not shown) due to the use of the bam function which is designed for large datasets^a^. The final model, Model X, can be written as:

$${} {\fontsize{9.6pt}{9.6pt}\selectfont{\begin{aligned} \textbf{Model X}: ~ \text{logit}(SMK) = b_{0} &+ \sum_{j=1}^{p} b_{j} Z_{j} + a_{0}(t) + a_{1}(t)age \\&+ a_{2}(t)\, alcohol, \end{aligned}}}  $$

where *Z*_*j*_ are the covariates with constant parameters *b*_*j*_, **a**(*t*)=(*a*_1_(*t*),*a*_2_(*t*)) are the time varying coefficients for the variables age and alcohol consumption respectively, and *a*_0_(*t*) is the time varying intercept.

The summary of the estimates for this model are shown in Table 3 with reported ORs and their 95% confidence intervals. All p-values which are significant at the 0.05 significance level are indicated in bold. However, one should not rely solely on the p-values for the splines as their computation tend to be underestimated [39], but the spline plots should also be considered for observing changes. As shown in Table 3, all the constant coefficients were found to be significant. In addition, the time varying coefficients for the age categories 18-29 and 40-49 as well as the do not drink alcohol category were found to be significant which can also be seen in the plots for these categories.

**Table 3:** **Summary of the smoking status varying coefficient model (Model X)**

**Variable**	**OR (95% C.I.)**	**p-value**
Age (Reference: 60-59)		
18-29	2.08 (1.91-2.25)	**<0.001**
30-39	1.79 (1.64-1.95)	**<0.001**
40-49	1.75 (1.61-1.89)	**<0.001**
50-59	1.46 (1.34-1.60)	**<0.001**
s(time):18-29	-	**0.003**
s(time):30-39	-	0.649
s(time):40-49	-	0.046
s(time):50-59	-	0.619
s(time):60-69	-	0.621
Sex (Reference: Female)		
Male	1.61 (1.58-1.64)	**<0.001**
Marital status (Reference: Married)		
Single	1.47 (1.44-1.51)	**<0.001**
Widowed or divorced	1.84 (1.78-1.90)	**<0.001**
Education (Reference: University		
or higher)		
High school	1.36 (1.32-1.39)	**<0.001**
Middle school	1.81 (1.75-1.87)	**<0.001**
Primary school or less	1.44 (1.38-1.51)	**<0.001**
Income (Reference: High)		
Medium	1.30 (1.27-1.32)	<0.001
Low	1.78 (1.73-1.83)	**<0.001**
Work status (Rerference: Works)		
Does not work	0.74 (0.73-0.76)	**<0.001**
Region (Reference: North)		
Centre	1.21 (1.19-1.24)	**<0.001**
South	1.10 (1.07-1.12)	**<0.001**
Physical activity (Reference: Active)		
Partially active	0.95 (0.93-0.97)	**<0.001**
Sedentary	1.15 (1.12-1.18)	**<0.001**
Alcohol consumption (Reference:		
High risk drinker)		
Low risk drinker	0.69 (0.64-0.75)	**<0.001**
Non-drinker	0.47 (0.43-0.52)	**<0.001**
s(time):High risk drinker	-	0.353
s(time):Low risk drinker	-	0.232
s(time):Do not drink	-	0.038
Depression (Reference:		
Not depressed)		
Depressed	1.43 (1.39-1.48)	**<0.001**

**Figure 1:**
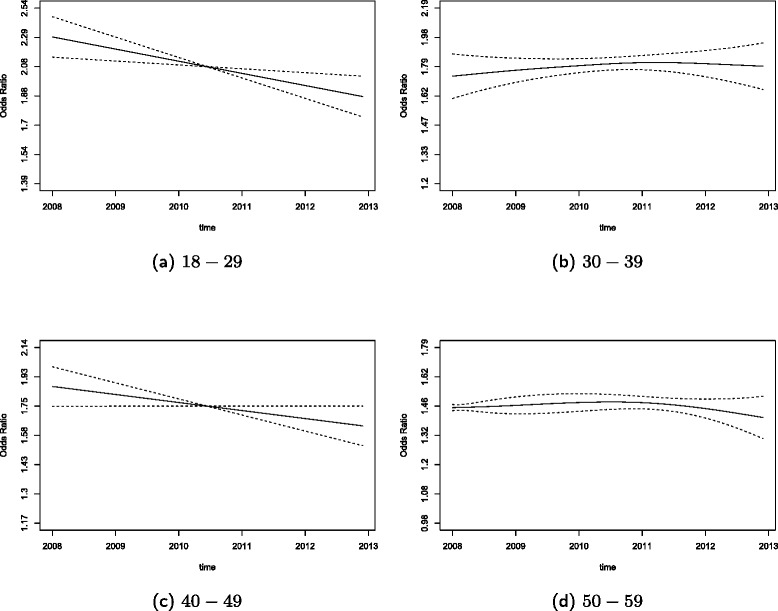
OR plots of the smoking VCM for the age categories.**(a)** 18-29, **(b)** 30-39, **(c)** 40-49 and **(d)** 50-59.

**Figure 2:**
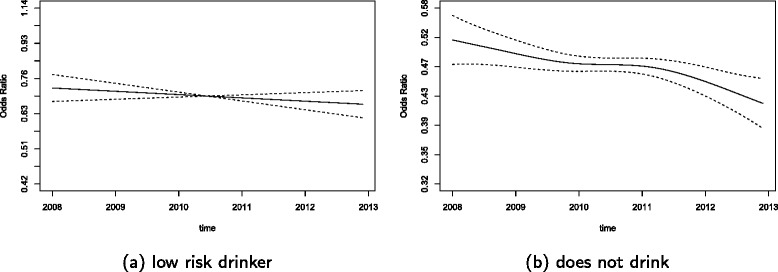
OR plots of the smoking VCM for the alcohol categories.**(a)** low risk drinker, and **(b)** does not drink.

To better visualize the change in the coefficients with time, odds ratio (OR) plots are produced. These plots are constructed by first adding the constant estimate of each category to the spline estimate of that category to obtain the overall effect, then taking the exponential, therefore producing plots on an exponential scale. This is conducted because the variables age and alcohol consumption in Model X have a constant coefficient found in *b*_*j*_ as well as the time varying coefficients found in *a*_*j*_(*t*). Odds ratio plots cannot be produced for the reference categories and therefore there are no plots for age 60−69 and the high risk drinker categories. For the remaining categories, OR plots are shown for the age categories in Figure 1 and the alcohol consumption categories in Figure 2. The age categories 18−29 and 40−49 both have a higher odds of being smokers than the reference age category of 60−69. However, as shown in the plots these odds are decreasing, indicating that the odds of being a smoker for these two age categories is decreasing in the period between 2008 and 2012. The other age categories of 30−39 and 50−59 were found to have a constant trend. The low risk drinker category for the alcohol variable showed a slow and not statistically significant decrease of the OR over time that remained below one. However, for the non-drinker category, there is a non-linear and significantly decreasing trend that is below an OR of one.

## Discussion

From this first application to this kind of data, the varying coefficient model, using P-spline estimation, appears to be a useful tool for evolutionary analysis using surveillance data The combination of the availability of big data such as the BRFS and a suitable sophisticated (for the epidemiological field) statistical approach is able to offer unique and important information in the public health field. This combination, at least from our analyses, seems to fulfil the search for evidence required in public health [40], particularly in understanding (and not just showing) the main changes in health outcomes and in health related risk factors. It highlights the major problems in the sub-populations of interest, and shows where and with whom interventions may be effective or may require more attention.

Another interesting aspect for both analysts and readers (epidemiologists, decision makers, etc.) is that this analytical approach returns an easily readable model as an output, showing what is influencing the outcome variable as well as what appears stable or changing over time. In addition, showing using OR plots the kind of change that has happened over time, without any linearity constraints, or any need to be monotonic. From a computational point of view, adopting the procedures proposed herein seems to be not too time consuming and quite straightforward, therefore showing promise for use in big data applications.

For the purpose of completeness and to further show how informative the approach can be, we will now comment on the specific results of the case examined in this application. Recent years have seen in Italy, as in many European countries, a steady and slow decline in the prevalence of smoking [41]. When, how, and where this decline is more evident are the fundamental questions to answer for evaluating past health promotion and public health action, and to drive future policies and interventions. Behavioural risk factor surveillance systems can offer abundant data to start to answer these and similar questions. In the case study presented, using the Italian surveillance PASSI, we have seen many potentially influential variables, such as gender, income or education, which appear to affect smoking prevalence in a stable manner over time, since these variables were found to have constant coefficients. Meaning that, for instance, actions aimed to reduce inequalities among these population strata in the five years of observation have not been successful, having the most deprived strata of the population odd ratios,all the other variables kept constant, significantly higher than those higher educated, higher income, etc.

On the other hand and as seen in the OR plots, some odds ratios have changed in recent years. For instance, the youngest age category and those who do not drink are smoking less compared to their respective reference categories. The preliminary results from the analysis is encouraging: in this five year period the younger generations seem to be smoking slightly less (decreases are significant, but still the ORs remain greater than one). This result is probably a combined effect of several interventions carried out in Italy in recent years, particularly targeted towards young people and school children. However, the analysis has shown that there is still much needed to be done to decrease smoking in other sub-populations. The small decrease of smoking prevalence observed in these years can be attributed to the fact that except for the youngest age group and non-drinker, those at higher risk do not appear to have changed their smoking behaviour. The poor, less educated, sedentary etc. present a prevalence of smoking higher than the others and these differences are constant over the years. What is worth noting is that the clustering of bad behaviours seems to be reinforced: those with a more risky drinking behaviour present an increasing prevalence of smokers (or, as shown in the graphs, those with less risky drinking behaviour present a decreasing prevalence of smoking). This could be an alarm bell for those working in the public health field, also showing possible tracks for interventions. If targeted interventions have been rather successful for some sub-populations, such as the young, more targeted interventions are needed also for other sub-populations that typically are more difficult to reach by general population interventions. Among the limitations of surveillance data, is the absence of a longitudinal component therefore the analysis is only able to show associations and not causal processes. Nevertheless, an analysis for sub-populations can be very informative as shown in this case. These results, obviously relevant for any policy analysis, have been possible through the proposed analysis although the time span of observation was only five years: it is possible that longer periods of observation could offer even more interesting results. In addition, it is worth noting that applying this method to health outcomes that need more time to change (both physically and behaviourally), such as some chronic diseases, may perhaps need a longer surveillance period to be able to observe interesting trends in the time varying coefficients.

## Conclusion

All these findings must be considered as preliminary results, since they are coming from a first application of this method to a single data system. Further analysis and application to other surveillance systems could provide further insight on how much this approach could result in a fundamental tool for a dynamic analysis of surveillance data. In addition, the present application does not include any interaction terms that may further affect the final results. Any significant interaction terms can be added beginning with a parametric logistic model before adding the varying coefficient terms. This was not performed in this case as the main purpose here was to demonstrate the usefulness of the method to BRFS data and not to present the most suitable model for smoking status in Italy.

Considering the limitations of this study, we can conclude that the application of the VCM techniques to BRFS data allows for the study of the changing effects of possible determinants on a health outcome/risk factor in order to better inform policy interventions.

As for the specific case studied, PASSI surveillance data analysed over time show how much there is still to do in order to produce a more relevant decrease of smoking prevalence in Italy. Some good signals are detected in the slow decrease in the OR of smoking among the youngest age category. However, no other sign of significant change has been observed. Major differences among population subgroups still exist indicating potential health inequalities and worrisome clustering of smoking behaviour with other negative behaviours such as risky alcohol drinking and sedentary behaviours.

## Endnote

^a^ Varying coefficient models can be estimated using the mgcv package in R software [42,43] and using the gam function that is used for fitting generalized additive models. However, since the data in the presented application is relatively large, to save computation time, the bam function of the package is used which works like the gam function but is designed for large datasets [43]. This can be especially useful in surveillance data analysis as longer periods of observation indicate very large and increasing sample size. When compared to the gam function, the bam function can take minutes to fit the most complicated model compared to several hours and even days depending on the sample size. For this function to perform even faster, the method used for selection of the smoothing parameter *λ* is by the fast REML computation method instead of the generalized cross validation method usually used by gam function. To use this function for estimating a varying coefficient model the “by” option is used as shown in the following example:

bam(SMK ∼Zj + INC + s(time, bs = ~ps~, k=55, m=c(3,2), by = INC), family = binomial(~logit~)),

where SMK is the response variable for smoking status, INC is the independent variable for income status, *Z*_*j*_ are all the other independent variables with constant coefficients, ps is for P-spline estimation, k=55 is the number of knots, and m=c(3,2) indicates the use of the third degree B-spline bases with a second order difference penalty.

For plots produced by the plot.gam function of the mgcv package, Bayesian confidence intervals are used for plotting of the smooth terms, which can be obtained by simulating from the posterior distribution of the functional coefficients (or varying coefficients) [39] For model selection, esting between nested models was performed using anova(model 1,model 2, test="Chisq") [39]. In addition the AIC of the models were found using the AIC function in R.
